# Involvement of brain cell phenotypes in stress-vulnerability and resilience

**DOI:** 10.3389/fnins.2023.1175514

**Published:** 2023-07-05

**Authors:** Cristiane Aparecida Favoretto, Marco Pagliusi, Gessynger Morais-Silva

**Affiliations:** ^1^Molecular and Behavioral Neuroscience Laboratory, Department of Pharmacology, Universidade Federal de São Paulo (UNIFESP), São Paulo, São Paulo, Brazil; ^2^Department of Pharmacology, Ribeirão Preto Medical School, University of São Paulo (USP), Ribeirão Preto, São Paulo, Brazil; ^3^Laboratory of Pharmacology, Department of Drugs and Medicines, School of Pharmaceutical Sciences, São Paulo State University (UNESP), Araraquara, São Paulo, Brazil

**Keywords:** stress outcomes, resilience, vulnerability, brain cell phenotypes, brain cell populations

## Abstract

Stress-related disorders’ prevalence is epidemically increasing in modern society, leading to a severe impact on individuals’ well-being and a great economic burden on public resources. Based on this, it is critical to understand the mechanisms by which stress induces these disorders. The study of stress made great progress in the past decades, from deeper into the hypothalamic–pituitary–adrenal axis to the understanding of the involvement of a single cell subtype on stress outcomes. In fact, many studies have used state-of-the-art tools such as chemogenetic, optogenetic, genetic manipulation, electrophysiology, pharmacology, and immunohistochemistry to investigate the role of specific cell subtypes in the stress response. In this review, we aim to gather studies addressing the involvement of specific brain cell subtypes in stress-related responses, exploring possible mechanisms associated with stress vulnerability versus resilience in preclinical models. We particularly focus on the involvement of the astrocytes, microglia, medium spiny neurons, parvalbumin neurons, pyramidal neurons, serotonergic neurons, and interneurons of different brain areas in stress-induced outcomes, resilience, and vulnerability to stress. We believe that this review can shed light on how diverse molecular mechanisms, involving specific receptors, neurotrophic factors, epigenetic enzymes, and miRNAs, among others, within these brain cell subtypes, are associated with the expression of a stress-susceptible or resilient phenotype, advancing the understanding/knowledge on the specific machinery implicate in those events.

## 1. Introduction

Since Hans Selye’s seminal letter to the editor published in Nature (Selye, 1936) describing the physiological responses to stress (he is also responsible for introducing this term in the biomedical field) and their role in homeostasis maintenance (Selye, 1946, 1956), thousands of studies were performed to clarify the role of stress exposure (especially chronic experience) in the development of diseases and the subjacent neural mechanisms. Over these 87 years, the neuroscience field went further with the classical hypothalamic–pituitary–adrenal (HPA) axis activation during stressful situations to uncover the role of specific neuronal populations on stress-related outcomes. Such advancements converged with the understanding of neurons as a functionally, morphologically, and genetically heterogeneous cellular population, with different subtypes playing specific roles in the functioning of our brains (Ecker et al., 2017). Thus, it is not surprising that many papers are now describing the role of specific neuronal populations on stress-related disorders and outcomes.

In this review, we will cover recent studies that, using different approaches, including Designer Receptors Exclusively Activated by Designer Drugs (DREADDs)-based chemogenetic, optogenetic, genetic manipulation, electrophysiology, pharmacology, and immunohistochemistry tools, explored the involvement of different neuronal phenotypes (as well as a brief discussion about the involvement distinct “functional states” of some non-neuronal brain resident cells) on stress-related responses and stress vulnerability versus resilience in preclinical models. It is not our intention to scrutinize literature looking for data regarding the relationship between every single brain cell subtype ever described and stress but to discuss the most relevant cell phenotypes for which studies are showing a possible role in stress-related disorders. For that, we used the terms “phenotype,” “subtype” or “subpopulation” for cellular populations that share functional, transcriptional, or electrophysiological characteristics within a brain region, and are related to a specific outcome (in many cases, outcomes).

We would like to highlight that discussing the “concept of stress” and the consequences of chronic stress exposure is out of the scope of this review. Nonetheless, this is a very interesting topic and we invite readers who wish to go further to look for the many excellent reviews from professor Bruce McEwen, Ph.D. In McEwen and Akil (2020), for example, the major concepts around stress, stress systems, and allostatic load are discussed.

## 2. Non-neuronal cells

**Figure 1:**
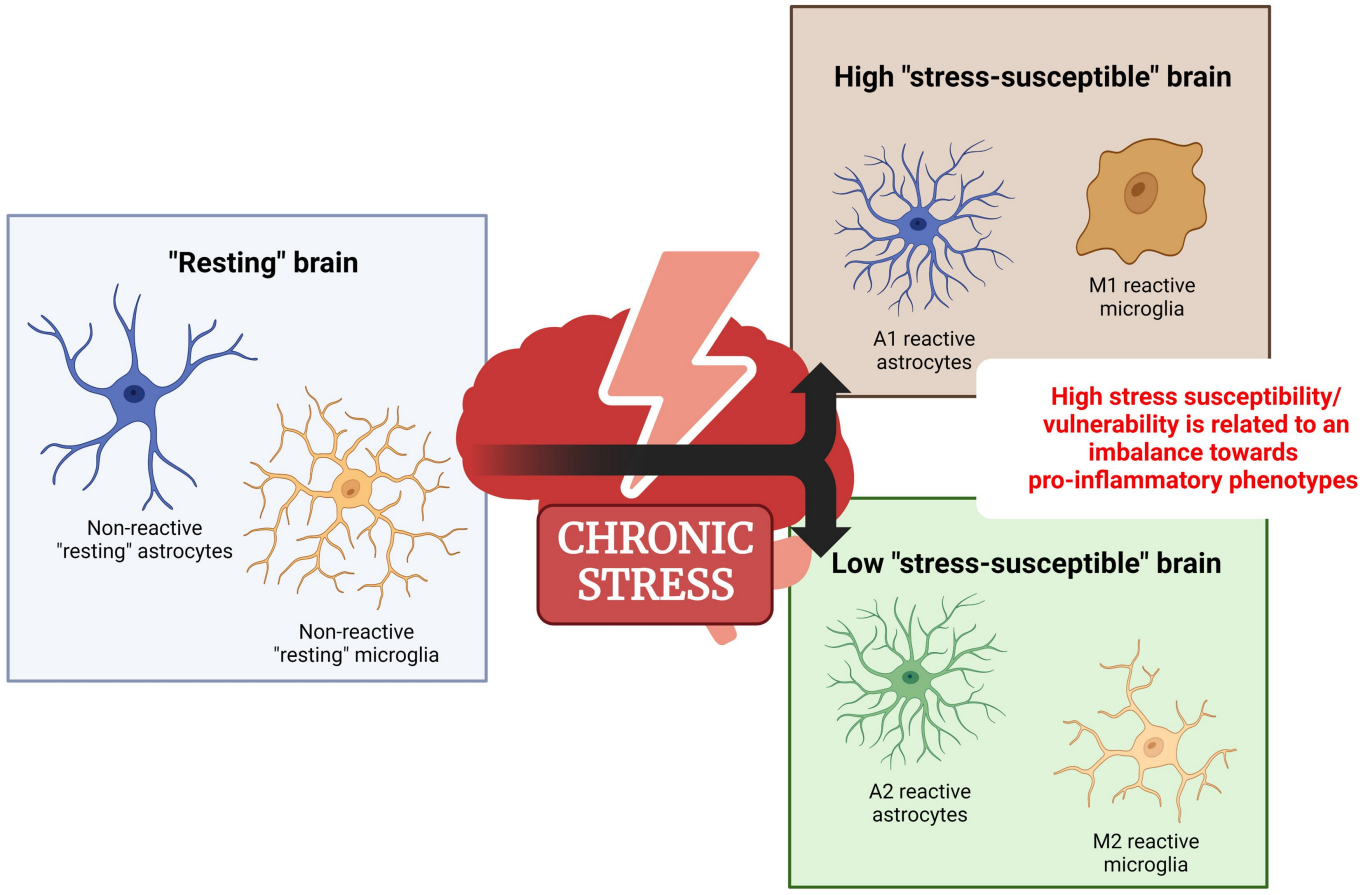
Dichotomous functional state of glial cells under chronic stress. In response to a noxious/adverse stimulus such as stress, astrocytes and microglia change from a non-reactive functional state to a reactive state. Evidence suggests that high-stress vulnerability should be linked to an imbalance toward a proinflammatory reactive phenotype. Created with BioRender.com.

Unlike neurons, glial cells, especially the astrocytes, and microglia, are highly dynamic, meaning that they rapidly change their functional state. This feature implies that these cells can exist in many functional states and change their phenotype depending on the stimuli. For instance, stress exposure is a powerful stimulus that promotes changes in glial cells’ functional state. Thus, here we will review the role of specific astrocyte and microglia functional states on stress-related disorders (Figure 1).

It is worth noting that there are a lot of studies looking into the relationship among peripheral immune responses, the central nervous system (CNS), and vulnerability to stress-related disorders. Results showed that the cytokines released by immune cells into the bloodstream reach the CNS to modulate neuronal and non-neuronal cell functioning, ultimately influencing behavior (Maes et al., 2009). For those interested in this topic, we invite the readers to look for the review from Cathomas et al. (2019) covering the aspects linking the immune system and stress vulnerability (Cathomas et al., 2019).

### 2.1. Astrocytes

The changing of astrocytes’ functional state in response to injury or noxious stimuli is called reactive astrogliosis. This phenomenon is highly dynamic and exists as a continuum, meaning that cells presenting different reactive phenotypes can be found co-existing in the same region (Anderson et al., 2014). Although it is still a matter of debate, recent literature classifies the reactive astrocytes into two different functional types, called A1 and A2 reactive astrocytes. This classification is based mainly on morphologic and transcriptomic analysis. In this context, the A1 reactive astrocytes show a pattern of upregulated genes related to inflammation and synaptic degradation, while the A2 reactive astrocytes have a set of genes upregulated that are related to neuronal survival, growth, and synaptic repair (Liddelow and Barres, 2017).

Early studies reviewed by Sofroniew (2009) showed a set of modulated genes and molecules in reactive astrocytes that later on were identified to be specific to each reactive astrocyte phenotype. In this context, LPS-reactive astrocytes, that possess a neurotoxic profile, showed a specific upregulation of genes involved in the expression of some components of the complement cascade (Zamanian et al., 2012), especially the secretion of the component C3 (Liddelow et al., 2017). Additionally, the upregulation of H2-D1 and Serping1 genes is found in the transcriptomic analysis of A1-reactive astrocytes (Zamanian et al., 2012; Liddelow et al., 2017; Fan and Huo, 2021). On the other hand, reactive astrocytes with a neuroprotective profile showed increased expression of Il-6, CLCF-1, and thrombospondins (Christopherson et al., 2005; Zamanian et al., 2012) and upregulated Ptx3, S100A10, and tweak receptor genes (Zamanian et al., 2012; Liddelow et al., 2017; Liddelow and Barres, 2017; Fan and Huo, 2021).

Recent data is showing a connection between the astrocytic A1-reactive phenotype and the consequences of chronic stress exposure. Astrocytes from the medial prefrontal cortex (mPFC) and hippocampus (HIP) of mice exposed to chronic social defeat stress (CSDS) showed a marked A1-transcriptional profile (including H2-D1 and Serping1 genes), meaning a probable increase in the neurotoxic A1-reactive phenotype (Hao et al., 2020). Moreover, astrocytes from B6.129S6-Il10tm1Flv/J homozygous mice, which have reduced IL-10 production and increased depressive-like behaviors after LPS treatment, are prone to an A1-specific phenotype in the cortex and HIP (Zhang et al., 2020). Obesity promotes A1-specific phenotype and is accompanied by increased susceptibility to CSDS. Moreover, the activation of astrocytes using a Gq-DREADD induced social avoidance and astrocytic A1-specific phenotype in resilient mice (Yu G. et al., 2022). The emergence of the A1-reactive astrocytes seems to precede the behavioral alterations in mice submitted to chronic mild stress (CMS) (Li S. et al., 2022). Finally, the chronic treatment with fluoxetine, a selective serotonin reuptake inhibitor clinically used as an antidepressant, inhibits the activation of A1-specific phenotype in the HIP and cortex of mice exposed to 6 weeks of chronic unpredictable stress (CUS), while improving the depressive-like behavioral alterations induced by stress exposure (Fang et al., 2022). This effect seems to be related to fluoxetine-induced activation of astrocytic 5HT-2B receptors.

It is worth mentioning that many studies found a reduction in GFAP expression in animal models of chronic stress exposure and post-mortem brains of depressed patients and linked such alterations to a reduced number of astrocytes. For instance, rats submitted to CUS showed decreased GFAP expression in HIP (Jiang et al., 2022), amygdala (Li Y. et al., 2022) and mPFC (Huang et al., 2022), similar to what is found in post-mortem studies using the brain of depressed patients (O’Leary and Mechawar, 2021). Thus, it seems that stress exposure has a dual effect on astrocytes, inducing the A1-reactive phenotype and reducing the number of non-A1-reactive cells. In this sense, astrocytes from mice exposed to chronic restraint stress (CRS) showed significant atrophy of their distal processes (Codeluppi et al., 2021), exhibiting a morphologic phenotype similar to astrocytes expressing an A1-reactive transcriptional profile (namely upregulated expression of C3 and Serping1 genes) (Althammer et al., 2020). Additionally, there is evidence that stress disrupts the blood–brain barrier (Welcome and Mastorakis, 2020), which properly functioning is highly dependent on astrocytes (Kadry et al., 2020).

The studies cited above did not evaluate possible sex differences in their experiments. The majority were performed in males while few did not report the sex used in the study. Thus, the results should be interpreted with caution since there are studies suggesting sex differences in reactive astrogliosis (Arevalo et al., 2013; Acaz-Fonseca et al., 2016).

### 2.2. Microglia

Being considered brain-resident macrophages due to their functional and transcriptional profile, the microglia play a vital role in brain homeostasis. Like astrocytes, they undergo numerous functional alterations in response to injury or noxious stimuli, a process sometimes called microglia activation (Woodburn et al., 2021). After activation, two cell phenotypes can be distinguished, coexisting in the same area, with different inflammatory profiles: proinflammatory (also called M1) or anti-inflammatory (also called M2) phenotypes (Jurga et al., 2020).

A set of transcriptional alterations and inflammatory factors release are often used to differentiate the activated microglia phenotypes. The M1 phenotype is characterized by the production of proinflammatory mediators such as the cytokines IL-1β, IL-6, IL- 12, IL-17, IL-18, IL-23, TNFα, IFNγ and chemokines CCL5, CCL20, CXCL1, CXCL9, CXCL10. Moreover, the production of iNOS, the inducible enzyme involved in nitric oxide synthesis, and the surface markers CD16 and CD32 are the most used markers to identify the M1 microglia. On the other hand, the M2 phenotype is characterized by the release of anti-inflammatory mediators, such as the cytokines IL-4, TGFβ, IL-10, and chemokines CCL2, CCL22, CCL17, and CCL24. However, the overexpression of Arginase 1 (Arg1) and the surface marker CD206 are the most used markers of M2 microglia (Boche et al., 2013; Jurga et al., 2020).

Early studies showed that corticosterone treatment and repeated restraint stress can induce microglia proliferation *in vivo* (Nair and Bonneau, 2006). Later on, many studies also reported increased microglia proliferation after submitting rodents to a variety of stressors in brain regions responsive to stress (Schramm and Waisman, 2022). This increase is blocked in mice deficient for the CX3CR1, an important receptor for the communication between neurons and microglia (Hellwig et al., 2016). Additionally, it has been shown that early-life stress alters microglia morphology and transcriptome in the developing HIP of mice (Delpech et al., 2016). At 14-days old, mice exposed to brief daily separation stress showed an increase in de-ramified microglial cells, and dysregulated expression of inflammatory genes at 28-days old, showing an increase in the expression of IL-6, Tnfrsf13b, and Ebi3, and reduced expression of inflammatory genes such as Lck, Tlr3, Tlr5, Tlr9, IL17ra, Notch1, Stat1, and Stat5, Il4ra, and Maf (Delpech et al., 2016). It is worthy of notice that such effects could be dependent on the brain region or stress protocol since there are data showing that exposure to CUS does not increase microglia number in the mPFC or the hypothalamic paraventricular nucleus (PVN) (Kopp et al., 2013).

Literature findings suggest that a shift toward a proinflammatory microglia phenotype can be related to stress consequences. The HIP and the mPFC of rats identified as susceptible to CMS showed increased expression of the proinflammatory markers IL-1β and IL-6 and up-regulation of molecules that mediate the microglia activation as CD11b, CX3CL1 and the CX3CR1 (Rossetti et al., 2016). Transcriptomic analysis of microglia isolated from susceptible mice to the CSDS revealed an increased expression of genes related to inflammation, phagocytosis, oxidative stress, and extracellular matrix remodeling (authors highlighted Lcn2, Mmp8, Mmp9, Hmox1, Hp, Gpx3, Cybb, Cd14, Myd88, and Ptgs2 transcripts), while the microglia from resilient mice showed an increased expression of genes related to cell plasticity to emotional stimuli (Cdk5r1, Egr3, Dnmt3a, FosB, JunD, Per2 transcripts) (Lehmann et al., 2018). C57BL/6J mice exposed to CMS showed increased Iba1 immunoreactivity in the dorsal HIP, indicating induction of microglia proliferation. However, this proliferation is bigger in animals identified as highly susceptible to this stressor (Zhang J. et al., 2021). In low-susceptible mice, there is an increase in Arg1-positive (Arg1+) microglia (Zhang J. et al., 2021), considered a neuroprotective cell phenotype (Jurga et al., 2020). The increase in Arg1+ microglia is related to wound healing, inhibition of inflammatory process, protection of the extracellular matrix, and homeostasis restoration, being the IL-4 cytokine known to act as an inductor of Arg1+ microglia phenotype (David and Kroner, 2011; Francos-Quijorna et al., 2016; Jurga et al., 2020). The overexpression of IL-4 in the HIP increases stress resilience and Arg1+ microglia and decreases IL-6 and IFN-γ upregulation in response to CMS, while the knockdown of its receptor (IL-4Rα) in this same brain region increases stress susceptibility, decreases Arg1+ microglia and increases TNF-α and IL-1β expression in response to subthreshold stress exposure (Zhang J. et al., 2021). In this work, authors discuss that such effects are related to an increase in brain-derived neurotrophic factor (*Bdnf*)-mediated HIP neurogenesis since IL-4 overexpression upregulated *Bdnf* expression, enhanced *Bdnf*-TrkB signaling and blocked the stress-induced decrease in HIP neurogenesis. The blockade of the *Bdnf*-TrkB signaling pathway also blocked IL-4-driven microglia effects on neurogenesis (Zhang J. et al., 2021). In line with those results, C57BL/6 J mice identified as susceptible to the CSDS showed increased Iba1 immunoreactivity in the dorsal HIP compared to resilient and control animals. Moreover, microglia from susceptible and resilient mice present distinct morphological changes: most microglia cells in the dorsal HIP of susceptible mice showed a de-ramified phenotype while in resilient mice hyper-ramified microglia were the majority (Fujikawa and Jinno, 2022).

Microglia activation seems to be also related to the increase in A1-reactive astrocytes in the HIP of mice exposed to CMS through the NF-kB pathway and Nod-like receptor protein 3 (NLRP3) inflammasome (Li S. et al., 2022).

Similar to studies involving astrocytes, the studies cited above did not evaluate possible sex differences in their experiments, even though studies suggest possible sex differences in microglia activation (Arevalo et al., 2013; Ocañas et al., 2023). In this sense, Bollinger and collaborators (2017) found that sex-differences in response to CRS exposure in some regions of the corticolimbic system. The CRS exposure increased iNOS, Arg1, and CX3CR1 expression in the orbitofrontal cortex of female rats, while increased only iNOS expression in the orbitofrontal cortex of males. The basolateral amygdala of female but not male rats showed increased expression of iNOS and decreased expression of Arg1 after CRS exposure, while the dorsal HIP of male rats showed increased iNOS and CX3CL1 expression after the CRS (Bollinger et al., 2017).

## 3. Neuronal cells

Going into the electrically excitable cells of the nervous system, here we will gather studies that actively manipulated the activity of different neuron subtypes (neuronal populations that share functional, transcriptional, or electrophysiological characteristics within a brain region) on animal models of chronic stress exposure or verified differences in specific cell phenotypes expression between susceptible and resilient subjects.

### 3.1. Medium spiny neurons

**Figure 2:**
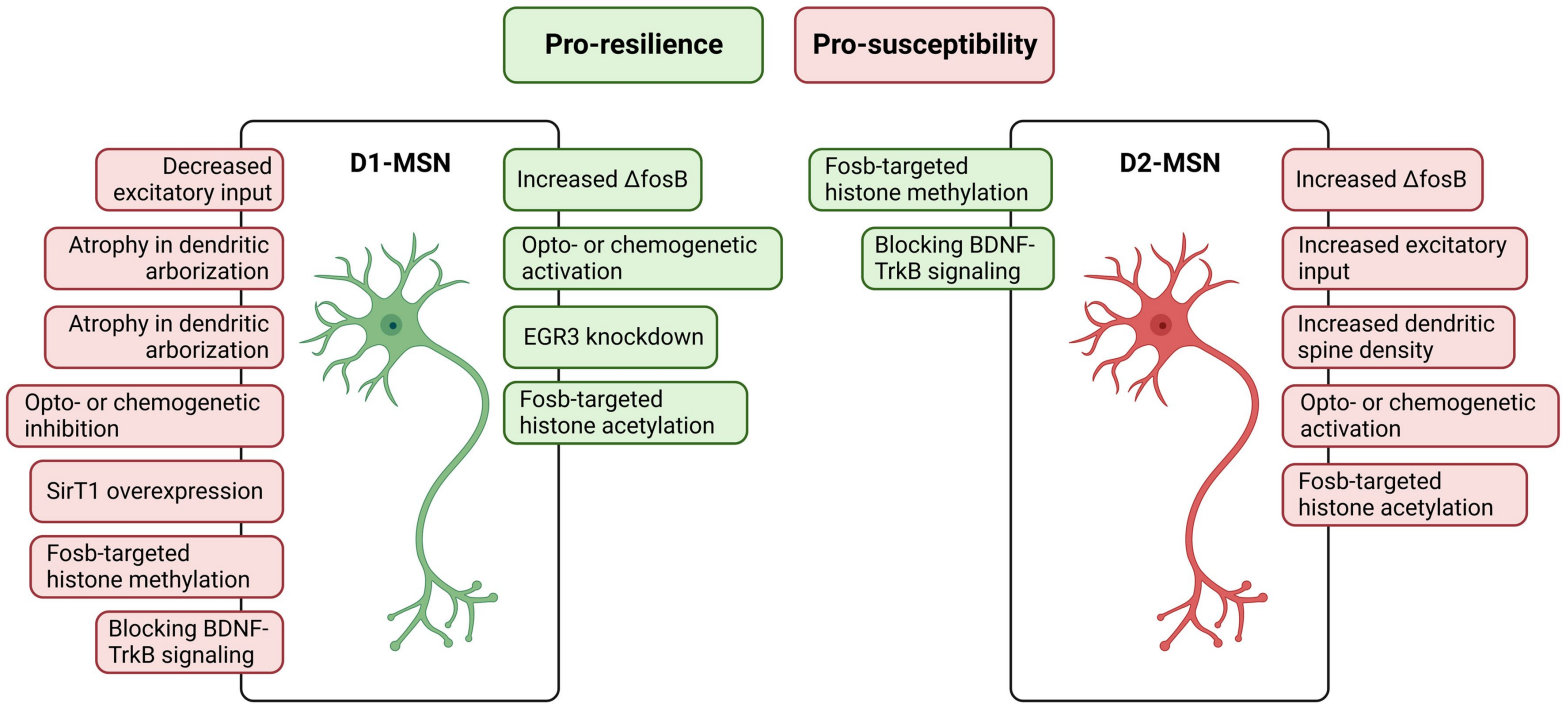
Features of nucleus accumbens D1- and D2-medium spiny neurons (MSNs) associated with resilience (in green) and susceptibility (in red). Generally, D1-MSNs pathway activation triggers a resilient phenotype and on the other hand, D2-MSNs pathway activation triggers a susceptible phenotype. Created with BioRender.com.

Medium spiny neurons (MSNs), although also occurring in other basal ganglia areas such as the amygdala, have been widely described and investigated in the dorsal and ventral striatum (which include the nucleus accumbens – NAc), being implicated in several disorders including depression, Huntington’s disease, and drug abuse (Francis and Lobo, 2017; Allichon et al., 2021; Bergonzoni et al., 2021; Liu et al., 2021). The MSNs are GABAergic neurons characterized by their specific expression of dopamine receptors D1 or D2, emerging two anatomically and functionally different MSNs subtypes named D1-MSNs and D2-MSNs (Gertler et al., 2008; Gangarossa et al., 2013). Despite the differential expression of the dopamine receptors (D1 or D2), both subtypes also differentiate from each other considering their output projections and other gene expressions (Gerfen et al., 1990; Lobo et al., 2006; Francis and Lobo, 2017). Because of these differences, both subtypes are engaged in different, commonly opposed, behavioral events and stress responses (Figure 2).

The CSDS paradigm has been used as a valuable tool to assess the NAc MSNs subtypes’ engagement in stress response. There are a set of studies indirectly showing that NAc MSNs activity regulates the stress response. Christoffel et al. (2011) showed an increase in the frequency of miniature excitatory postsynaptic currents after CSDS. Lobo et al. (2013) showed that ΔFosB – a proxy for chronic neuronal activation – is specifically increased in NAc and dorsal striatum D2-MSNs of mice susceptible to CSDS and, interestingly, showed that ΔFosB is increased in NAc and dorsal striatum D1-MSNs of mice resilient to CSDS. Additionally, ΔFosB in the NAc is known to mediate the antidepressant action of drugs and voluntary exercise (Vialou et al., 2010; Mul et al., 2018).

Prompted by these findings, Francis et al. (2015) demonstrated, using opto- and chemo-genetics, that NAc D1-MSNs activation induced a resilient-like stress outcome, while NAc D1-MSNs inhibition or NAc D2-MSNs activation facilitate susceptible-like stress outcomes. The authors also demonstrated that NAc D2-MSNs show an increase in the frequency of excitatory synaptic input, while NAc D1-MSNs show a decrease in this electrophysiological property in susceptible mice to CSDS. Further investigations using fiber photometry calcium imaging in freely moving mice demonstrated that D1-MSNs activity predicts susceptibility to stress, i.e., increased baseline on D1-MSNs activity prior to stress is found in mice resilient to CSDS (Muir et al., 2018). Overall, the NAc D1-MSNs pathway triggers a resilient phenotype when stimulated, protecting the individual from stress. On the other hand, the NAc D2-MSNs pathway when stimulated triggers a susceptible phenotype, facilitating behavioral impairments induced by chronic stress.

There are also structural alterations observed in D1- and D2-MSNs following chronic stress. Despite not differentiating cell subtypes, Bessa et al. (2013) showed MSNs hypertrophy in the NAc and increased NAc expression of *Bdnf*, neural cell adhesion molecule 1 (*Ncam1*), and synapsin 1 (*Syn1*) – all known to be related to neuroplasticity - of rats displaying stress-induced anhedonia behavior, a core symptom of depression. Also, NAc MSNs have smaller postsynaptic densities and increased IκB kinase (IKK) – a molecule directly associated with neuronal morphology regulation, besides being critical for broad cell functions – after CSDS (Christoffel et al., 2011). Regarding MSNs subtypes, D2-MSNs on NAc showed increased dendritic spine density after CSDS, a structural change correlated with social avoidance behavior (Fox et al., 2020b). On the other hand, despite no changes were found in dendritic spine density, there was atrophy in NAc D1-MSNs dendritic arborization in mice susceptible to CSDS (Francis et al., 2017; Fox et al., 2020a). This CSDS-induced NAc D1-MSNs atrophy is regulated by early growth response 3 (EGR3) and GTPase RhoA (Francis et al., 2017, 2019; Fox et al., 2020a). The first is a transcription factor expressed in response to neuronal activation, being downstream of major intracellular cascades associated with neuronal plasticity (Li et al., 2007; Chandra and Lobo, 2017). The latter is a member of the Rho family which are key regulators of synaptic plasticity (Zhang H. et al., 2021). Specific EGR3 knockdown on NAc D1-MSNs restored the CSDS-induced atrophy in this cell subtype, also reversing the behavioral impairments (Francis et al., 2017). Similarly, knockdown or pharmacologic inhibition of RhoA prevents CSDS-induced depressive outcomes by preventing D1-MSN atrophy (Francis et al., 2019; Fox et al., 2020a).

Based on this, it is evident that MSNs plasticity is a key regulator controlling stress response. The *Bdnf*, along with the above-mentioned and other molecules, is also associated with cellular plasticity and has been implicated in several stress-related disorders (Lin and Huang, 2020). The *Bdnf* pathway, which is mediated by its receptor tyrosine kinase B (TrkB), in the NAc MSNs is unsurprisingly involved in the stress response. It is known that increased *Bdnf* in the NAc is associated with stress susceptible outcomes, and intra-NAc *Bdnf* infusion increases susceptibility to social stress (Berton et al., 2006; Krishnan et al., 2007; De Vry et al., 2016; Junior et al., 2018). Prompted by these findings, Pagliusi et al. (2022) demonstrated that the *Bdnf*–TrkB signaling in D2-MSNs mediates susceptibility to CSDS and, in contrast, *Bdnf*–TrkB signaling in D1-MSNs is associated with stress resilience. These mechanisms were investigated in both, male and female mice.

Beyond the neuronal plasticity are epigenetics alterations, which have been also implicated in the MSNs in stress response. Kim et al. (2016) showed that SirT1 – a protein linked with genomic complexity through its deacetylase action – in the NAc mediates stress response. The overall overexpression of SirT1 in the NAc promotes susceptibility to stress and, inversely, selective ablation of SirT1 in the NAc promotes resilience to stress (Kim et al., 2016). Regarding MSNs subtypes, overexpression of SirT1 in NAc D1-MSNs induces susceptibility to stress, with no effect when SirT1 is overexpressed in NAc D2-MSNs (Kim et al., 2016). Similarly, Fosb-targeted histone post-translational acetylation in D2-MSNs or methylation in D1-MSNs promotes stress susceptibility and, oppositely, histone methylation in D2-MSNs or acetylation in D1-MSNs promotes stress resilience (Hamilton et al., 2017). Finally, dimethylation of histone H3 and its controlling enzymes are enriched in NAc D2-MSNs and are involved in the stress susceptibility induced by early life stress, this effect seems not to be displayed in D1-MSNs (Kronman et al., 2021).

NAc D1- and D2-MSNs also play an important and opposite role in sleep regulation, with an impact on CSDS outcomes. Insomnia and depression are highly overlap medical conditions with depressed patients often exhibiting altered sleep patterns. Moreover, insomnia is a strong independent predictor of depressive disorders and suicide (Riemann et al., 2020). While NAc D1-MSNs are exclusively involved in rapid eye movement (REM) sleep, NAc D2-MSNs are involved in slow-wave sleep (McCullough et al., 2021). CSDS may affect sleep regulation by increasing the time spent in REM and slow wave sleep in addition to decreasing time spent awake, which seems to be mediated by a specific increase in cAMP response element-binding protein (CREB) within NAc D1-MSNs (Wells et al., 2017; McCullough et al., 2021). Interestingly, NAc D1-MSNs inhibition along with NAc D2-MSNs activation resembles the CSDS impairment on sleep (McCullough et al., 2021).

### 3.2. Parvalbumin neurons

The neuronal subpopulation which expresses parvalbumin (PV), a calcium-binding protein, is present in brain areas vastly implicated in responses to stressful stimuli and stress susceptibility and resilience mechanisms, including the cerebral cortex and HIP, that will gain special focus in the present section of this review. In the CNS, the majority of PV-positive cells are interneurons, while a small percentage of this neuronal subpopulation are projection neurons (Celio, 1990; Zaletel et al., 2016). PV interneurons are connected by gap junctions and present a high level of firing synchronicity. Due to these electrophysiological properties, they are also frequently called fast-spiking neurons and exert crucial modulatory action on other projection neurons (Kawaguchi et al., 1987; Traub et al., 2001; Damodaran et al., 2014).

Research has pointed to the participation of the cerebral cortex PV cell subpopulation in resilience to stress: reduction of PV neurons activity or expression has been positively associated with the emergence of stress-related behavioral symptoms (Perova et al., 2015; Li X. et al., 2022). For example, chemogenetic inhibition of PV interneurons in the whole mPFC and in the infralimbic (IL) mPFC produced, respectively, helplessness behavior (Perova et al., 2015), and enhanced aggressive behavior combined with decreased social preference (Li X. et al., 2022). Consistently, activation of IL PV interneurons reduced stress-induced social investigation deficits (Li X. et al., 2022). Moreover, susceptible subjects to chronic stress exhibited a reduced number of PV interneurons in the primary motor cortex (Serradas et al., 2022) and IL mPFC (Czéh et al., 2018), paralleled with stress-induced behavioral impairments.

Although the barrel cortex, a region of the somatosensory cortex, is a relatively little studied brain area in the neurobiological consequences of the stress research field, the work by Chen et al. (2018) revealed that the activity of barrel cortex inhibitory PV interneurons plays important role in stress resilience. Exposure to restraint stress reduced activation levels of barrel cortex PV interneurons, enhanced dendritic spine elimination in layer 5 pyramidal neurons in this region, and produced recognition memory deficits. Chemogenetic activation of barrel cortex PV interneurons during stress exposure successfully prevented stress-induced behavioral impairment and layer 5 pyramidal neuron spine loss (Chen et al., 2018). In agreement with those findings, repeated restraint stress also led to dendritic spine loss in the frontal association cortex of mice, an impact that was attenuated by treatment with ketamine, a glutamatergic NMDA receptor antagonist (Ng et al., 2018). Compelling results also demonstrated that ketamine treatment enhanced the activity of frontal association cortex PV interneurons in stressed subjects and, in the same way, chemogenetic activation of these cells prevented stress-produced spine loss (Ng et al., 2018).

Deepening our discussion, some studies have shed light on specific molecular mechanisms underlying the involvement of prefrontal cortex PV neuronal subpopulation on vulnerability and resilience to stress. In humans, mutations in the oligophrenin-1 (OPHN1) gene - which encodes for a Rho-GTPase-activating protein – have been linked with several symptoms, including intellectual disability and behavioral disorder (Santos-Rebouças et al., 2014; Schwartz et al., 2019). Accordingly, selective deletion of OPHN1 in prelimbic (PL) mPFC PV interneurons promoted helplessness behavior in mice (Wang M. et al., 2021). Also, the blockage of the RhoA/Rho-kinase pathway as well as chemogenetic activation of PL PV interneurons was able to reverse this depressive-like behavior in PV-OPHN1 knockout mice. Moreover, another study found that intra-mPFC treatment with a peptide that prevents NMDA receptor subunit NR2A association with the postsynaptically expressed PSD-95 protein reduced the anxiety-like behavior and PL PV interneuron loss produced by a history of early-life maternal separation stress, in adolescent rats (Ganguly et al., 2015). Of note, the NR2A subunit seems to have crucial participation in the maturation of cerebral cortex PV interneurons (Zhang and Sun, 2011).

Taken together, the studies presented so far indicate that reduced activity and expression levels of PV neurons, in different cerebral cortex subareas, seem to be associated with the emergence of stress-related behavioral and neurobiological outcomes, while activation of these cells in specific cerebral cortex subregions led to a stress-resilient phenotype. However, other studies point in the opposite direction, where the increased activity of mPFC PV cells is associated with stress susceptibility. Moreover, those works also importantly demonstrate that the role of the mPFC PV neuronal population in stress resilience versus susceptibility seems to vary in a sex-dependent manner.

In this regard, Page et al. (2019) found that chronic unpredictable mild stress (CUMS) enhanced the activation of PV-positive cells in the mPFC of both male and female mice. However, repeated chemogenetic activation of PV cells in this brain area increased anxiety-like behaviors of female, but not male mice, in the open field and novelty-suppressed feeding tests (Page et al., 2019). Also, Shepard and Coutellier (2018) reported that female mice exhibited graver behavioral consequences of exposure to CUMS relative to males. In the same way, only stressed females presented increased numbers of PV neurons in the mPFC, and the altered prefrontal expression of synaptic markers of glutamate transmission on PV cells also obeyed a sex-dependent fashion (Shepard and Coutellier, 2018). Therefore, further studies must be carried out to help unravel the complex mechanisms underlying vulnerability and resilience to stress involving the subpopulation of PV neurons in specific subregions of the cerebral cortex.

Advancing our review, now we will present studies that investigated the involvement of hippocampal PV cell subpopulation in stress vulnerability and resilience to stress and see how it compares to the previously discussed participation of the cerebral cortex PV neurons in these processes. In the HIP, PV GABAergic interneurons exert important action in neuroplasticity associated with memory and learning and are particularly susceptible to repeated exposure to stress (Zaletel et al., 2016; Nahar et al., 2021). Despite their relevant role in coordinating hippocampal activity, the PV cells represent a small percentage of the entire hippocampal population (Freund and Buzsáki, 1998).

Chemogenetic inhibition of PV neurons of the hippocampal dentate gyrus (DG) induced social interaction deficits in mice submitted to a subthreshold social defeat stress (SSDS) - not capable of inducing social avoidance behavior *per se* (Medrihan et al., 2020). Exploration of molecular targets involved in those responses found that selective deletion of the p11 protein, a calcium sensor implicated in the effects of antidepressant drugs, in PV neurons diminished the firing frequency of DG PV cells and led to social investigation impairment (Medrihan et al., 2020). Potassium channel kv3.1 levels, regulated by the p11, seem to be a mechanism underlying those described effects. Markedly, kv3.1 overexpression in DG PV neurons produced a resilient phenotype in mice exposed to CSDS.

In contrast to those findings, activation of PV cells from the ventral DG following SSDS culminated in stress susceptibility, and the inhibition of such neurons increased social interaction and preference for sucrose solution in socially defeated mice (Bhatti et al., 2022). Also, the knockout of the Ahnak gene in PV cells of the ventral DG produced resilience to CSDS (Bhatti et al., 2022). Very interestingly, Ahnak regulates L-type voltage-gated calcium channels in PV interneurons and seems to interact with the aforementioned p11 protein, exhibiting important modulatory tone on depressive-like behaviors (Jin et al., 2020; Mantas et al., 2022).

Regarding the expression levels of PV cells in HIP subregions among non-stressed, stress-susceptible, and stress-resilient subjects, it was found fewer PV interneurons in DG, CA1, and CA2-3 hippocampal subregions of the dorsal HIP, and in the CA1 subarea of the ventral part of the hippocampal formation in both anhedonic and resilient animals to CMS relative to controls (Czéh et al., 2015). However, contrary to those findings, Nieto-Gonzalez et al. (2015) did not observe alterations in the number of PV interneurons in the DG of control, susceptible, and resilient rats to CMS.

Therefore, as in the cerebral cortex, it is critical that the hippocampal PV cell subpopulation receives further attention to build an understanding of the exact molecular machinery involved in stress vulnerability and resilience processes within this neuronal subtype. Also, it is essential to explore whether and/or how those mechanisms depend on the studied brain subregions, type, and duration of stress protocol, characteristics of experimental subjects (sex, age, species), PV modulation on specific neuronal circuits, and stress-related outcomes, among others.

It is important to emphasize that besides the aforementioned brain regions, the PV neuronal subpopulation that composes other key areas implicated in stress responses also seems to participate in the cellular and molecular mechanisms that underlie vulnerability or resilience to stress. For example, inhibition of ventral pallidum (VP) PV neuronal activity prior to exposure to CSDS promoted a resilient phenotype (Knowland et al., 2017). Moreover, separate neural pathways seem to control the expression of different stress-induced depressive-like behaviors: inhibition of PV neurons projecting from VP to the ventral tegmental area (VTA) specifically reversed stress-provoked social interaction deficits, while inactivation of VP PV cells projecting to lateral habenula only reduced the despair-like behavior presented by stress susceptible mice (Knowland et al., 2017). Still, regarding the VP neuronal subpopulations, the Npas1 (Neuronal PAS 1) protein-positive (Npas1+) neurons also seem to be implicated in the vulnerability to chronic stress exposure. Recent work showed that the chemogenetic inhibition of the VP Npas1+ neurons enhanced resilience to CSDS and chronic witness defeat stress (CWDS) in mice, while the activation of hM3Dq receptors in this same neuronal phenotype increased vulnerability to SSDS (Morais-Silva et al., 2023). Furthermore, Regev-Tsur et al. (2020) found increased activation of PV interneurons in the basolateral amygdala of rats vulnerable to juvenile stress combined with underwater trauma, relative to resilient and control subjects.

**Figure 3:**
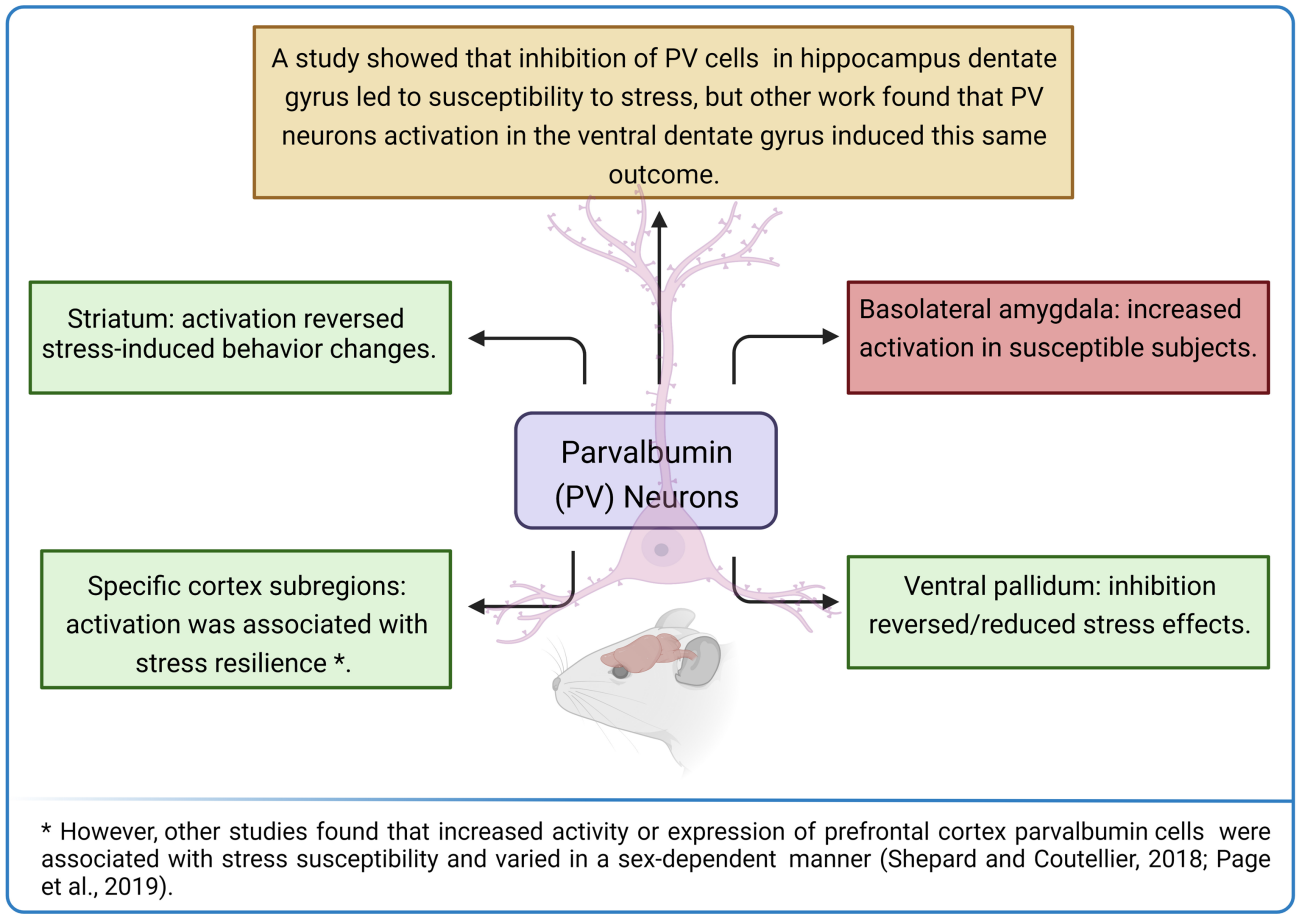
Activity and expression levels of parvalbumin (PV) expressing neurons seem to be associated with stress-resilient or susceptible phenotypes depending on the evaluated brain region. Created with BioRender.com.

However, in a different sense of those results, optogenetic activation of striatal PV interneurons reversed the impairment in decision-making behavior that followed exposure to chronic stress, while optogenetic inhibition of these neurons mimicked the stress effects in control animals (Friedman et al., 2017; Figure 3). Therefore, PV activity and expression levels seem to associate with resilience or susceptibility to stress depending on the studied brain region, circuit, and possibly on other factors, as already mentioned (characteristics of stressor, evaluated stress-related effect, experimental subject, among others).

To close this topic, it is interesting to mention the significant role of the perineuronal nets (PNNs) that preferentially enwrap inhibitory interneurons, including PV-positive cells, in stress consequences. The PNNs are components of the extracellular matrix that circumvent cell bodies, proximal dendrites, and initial portions of axons. The PNNs seem to protect the cell against oxidative stress, it is important for the settlement of its fast-spiking electrophysiological properties, and stabilization of synapses, and are associated with the termination of the neuroplasticity period (Cabungcal et al., 2013; Brewton et al., 2016; Fawcett et al., 2019). Manipulations that decreased PL PNNs expression during adolescence reduced stress resilience in adult life (Yu Z. et al., 2022). In addition to opposing depressive-like symptoms and altered function of PV neurons, treatment with ketamine also enhanced PNNs expression levels in the PL mPFC (Yu Z. et al., 2022). Beyond that, indicating a possible molecular mechanism of how stress affects PV neurons PNNs leading to vulnerability to neuropsychiatric disorders, the work by Page et al. (2018) showed that exposure to chronic stress during adolescence increased the number of mPFC PV neurons involved by PNNs. However, stressed mice exhibiting deficiency of the transcriptional factor Neuronal PAS 4 (Npas4), did not exhibit this alteration. In parallel, Npas4 heterozygous mice submitted to stress presented an impaired performance in a cognitive flexibility task, relative to the effects of stress or genotype alone (Page et al., 2018). Therefore, although we did not encounter a study providing causal evidence that modulation of PV cells PNNs, specifically, produces susceptibility or resilience to stress, some works indicate its potential involvement in those mechanisms that are worth further exploring.

### 3.3. Pyramidal neurons

The pyramidal neurons are a class of multipolar excitatory neurons, highly preserved across species, that are distributed through forebrain regions related to the executive, cognitive, and emotional functions (Sugino et al., 2006; Spruston, 2008). Thus, it is not surprising that many studies have shown that chronic stress exposure alters pyramidal neuron morphology and activity, implicating in disrupted functioning across stress-related brain regions, such as the mPFC, HIP, and amygdala (McEwen et al., 2016). For example, chronic stress exposure is related to dendritic alterations of pyramidal neurons, as chronic restraint or psychosocial stress exposure induces apical dendritic atrophy in CA3 pyramidal neurons (McEwen, 1999), repeated SDS induces dendritic atrophy in CA1 pyramidal neurons (Patel et al., 2018), chronic restraint or immobilization stress induces dendritic atrophy in mPFC pyramidal cells (McEwen and Morrison, 2013) and chronic immobilization, chronic restraint stress or repeated SDS induces dendritic arborization growth in the basolateral amygdala (Vyas et al., 2002; Patel et al., 2018; Blume et al., 2019).

Two elegant studies identified pyramidal neurons in the mPFC (using a morphologic characterization of the genetic specificity of the CAMKII promoter) and evaluated the effect of their activation on stress-induced behavioral alterations. Kumar et al. (2013) optogenetically activated the pyramidal neurons in the Layer V of the PL mPFC for 14 days after the CSDS. The repeated optogenetic activation of the PL Layer V pyramidal neurons blocked the stress-induced anxiogenic responses in the elevated plus maze (EPM) (Kumar et al., 2013). Similarly, the chemogenetic activation of the mPFC pyramidal neurons reversed the increased aggressiveness in the resident-intruder test and memory deficits in the novel object recognition test induced by CUS exposure (Wei et al., 2018). This improvement was related to the restoration of the glutamatergic neurotransmission within the mPFC and GABAergic neurotransmission in the basolateral amygdala (Wei et al., 2018).

Unlike the two studies cited above, most of the studies rely only on the specificity of the CAMKII promoter (Sugino et al., 2006; Wang et al., 2013) to manipulate the activity of the pyramidal neurons in neocortical structures. In this sense, the optogenetic inhibition of the CAMKII positive mPFC neurons projecting to the dorsal raphe nucleus decreased the stress-induced social avoidance (Challis et al., 2014), while the activation of the CAMKII positive mPFC neurons projecting to the NAc reverted social avoidance and sucrose preference decrease in SDS susceptible mice (Vialou et al., 2014). On its turn, the optogenetic activation of the excitatory neurons projecting from the mPFC to the basolateral amygdala blocked the increase in anxiety-like behaviors in stress-susceptible mice (Vialou et al., 2014; Yu H. et al., 2022). Such results suggest that the effects of the experimental manipulation of the pyramidal neurons activity in the mPFC are highly dependent on the projection targets examined. Additionally, the specific optogenetic activation of the excitatory neurons on the left hemisphere of the mPFC inhibits CSDS-induced social avoidance in mice (Lee et al., 2015).

The mPFC pyramidal neurons of female and male rats seem to be differentially affected by stress exposure and distinct projections regulate stress consequences (Wellman et al., 2018; Tan et al., 2021). Tan and collaborators (2021) found that chronic isolation stress induces distinct behavioral alterations in male and female mice: male mice showed heightened aggressive behavior while female mice showed decreased social approach. In males, the chemogenetic activation of the excitatory mPFC neurons projecting to the basolateral amygdala rescued the behavioral deficits while in females the chemogenetic activation of the excitatory mPFC projections to the ventral tegmental area reverted social deficits after social isolation stress (Tan et al., 2021).

Two important mechanisms of neuronal plasticity that pyramidal cells undergo, and are extensively studied in the HIP, are the long-term depression (LTD) and long-term potentiation (LTP) of their activity (Spruston, 2008). Nonetheless, it is not surprising that chronic stress exposure affects such mechanisms (Kim and Diamond, 2002). In this sense, the attenuation of the postsynaptic activity in the excitatory inputs from the ventral HIP to the NAc through the induction of LTD by low-frequency optogenetic stimulation of the excitatory neurons (likely pyramidal cells) has a pro-resilience effect in mice exposed to the CSDS (Bagot et al., 2015), while the acute activation of such projections increases the CSDS-induced social avoidance (Bagot et al., 2015). On the other hand, the optogenetic activation of the CA3 CAMKII positive neurons from the dorsal HIP projecting to the dorsolateral septum decreases CSDS-induced social avoidance in the social interaction test and immobility in the forced swim test (FST) (Wang H. et al., 2021). The chemogenetic activation of the pyramidal neurons of the dorsal HIP also blocked the increase in locomotor activity induced by CUS exposure in rats (Wei et al., 2018). Recently, Cole et al. (2022) showed that the stimulation of the excitatory CA1 neurons of the ventral HIP projecting to the bed nucleus of the stria terminalis and to neuropil surrounding the paraventricular nucleus of the hypothalamus decreases the corticosterone response to an acute stressor (Cole et al., 2022).

Literature findings indicate that an overactivated amygdala (especially the basolateral amygdala) functioning is related to chronic stress effects and mood disorders (McEwen et al., 2016). In this sense, the chemogenetic inhibition of the basolateral amygdala excitatory neurons decreased the heightened aggressiveness in rats exposed to the CUS (Wei et al., 2018), while the optogenetic activation of excitatory BLA neurons projecting to the mPFC (Felix-Ortiz et al., 2016; Lowery-Gionta et al., 2018) and to the ventral HIP (Felix-Ortiz et al., 2013) in stress-naïve mice recapitulates the stress-effects in the EPM. The activation of such neurons did not change stress-increased anxiety-like behaviors in the EPM (Xiao et al., 2020). As for mPFC and HIP, the studies manipulating the pyramidal neurons in the amygdala rely on the specificity of the CAMKII promoter to use the chemogenetic and optogenetic approaches (Sugino et al., 2006; Wang et al., 2013).

### 3.4. Serotonergic neurons

Serotonergic neurons project mainly from the dorsal and median raphe nuclei and are highly vulnerable to stress exposure, being vastly implicated in stress-related neuropsychiatric disorders, including depression and anxiety (Hale et al., 2012; Pollano et al., 2018). Here, we will present research that demonstrates how serotonergic neurons also seem to have an important role in stress susceptibility and resilience mechanisms.

Rats classified as non-responsive to submersion stress displayed an increased number of serotonergic cells in the dorsal raphe nucleus (DRN) relative to control and stress-responsive (vulnerable) subjects (Adamec et al., 2012). Also, female monkeys classified as sensitive to a protocol that combined exposure to metabolic and psychosocial stressors exhibited a reduced number of DRN serotonergic cells relative to highly-stress resilient individuals (Bethea et al., 2005). Moreover, the activity of a specific serotonergic neuron subpopulation was associated with resilience to stress. Chronic optogenetic inhibition of serotonergic neurons projecting from the DRN to VTA induced social interaction deficits in mice previously categorized as resilient to CSDS. Additionally, acute or chronic stimulation of these neurons reversed the social interest impairments in previously susceptible subjects to CSDS, therefore promoting a resilient phenotype (Zou et al., 2020). Those findings indicate that increased serotonergic neuron expression or activity is associated with stress resilience. However, pointing in the opposite direction, another work verified that susceptible individuals to CSDS exhibited an increased number of serotonergic neurons in the ventral subnucleus of the DRN relative to control and resilient subjects (Prakash et al., 2020).

Research has demonstrated the involvement of diverse molecular mechanisms within serotonergic neurons in stress susceptibility and resilience, shedding light on possible targets for the treatment of stress-induced behavioral, physiological, and neurobiological consequences. Among those are neurotrophic factors, microRNAs, protein kinases, epigenetic enzymes, receptors, and others, as we will discuss below.

Selective deletion of *Bdnf* in serotonergic neurons induced depressive-like behaviors, including a reduced preference for sucrose solution and increased immobility in the FST in female, but not male, mice submitted to subchronic unpredictable stress, which does not induce behavioral changes *per se* (Meng et al., 2020). In males exposed to subchronic stress, ablation of *Bdnf* from serotonergic neurons specifically reduced the duration of female urine sniffing, indicating decreased sex-related reward-seeking behavior (Meng et al., 2020). In agreement with those findings, another study reported that overexpression of *Bdnf* in serotonergic neurons increased serotonergic axonal fiber volume and length, and adult neurogenesis in hippocampal DG, as well as enhanced contextual fear memory behavior. Furthermore, serotonergic neuron *Bdnf* overexpression reduced the depressive-like behavioral impacts of exposure to CSDS (Leschik et al., 2022).

MicroRNAs are small noncoding RNAs that control gene expression (Hammond, 2015). Research demonstrated that antidepressant treatment enhanced microRNA-26a-2 (miR-26a-2) levels in the DRN and that miR-26a-2 downregulates the expression of 5-HT1A autoreceptors in serotonergic neurons. Remarkably, overexpression of miR-26a-2 in serotonergic neurons produced a resilient phenotype to CSDS, which was abolished by overexpression of 5-HT1A (Xie et al., 2019). In the same way, antidepressant treatments increased microRNA 135a (miR135a) levels in raphe nuclei, and overexpression of miR135a in serotonergic neurons also induced resilience to CSDS. Moreover, overexpression of miR135a in serotonergic neurons reduced baseline serotonin levels, increased baseline serotonin metabolism, and blocked the stress-induced decrease in serotonin levels in subareas of HIP, amygdala, raphe nuclei, and mPFC (Issler et al., 2014).

Genome-wide association studies (GWAS) linked variations in the CACNA1C gene, which encodes for the alpha-1c subunit of Cav1.2 L-type calcium channels, with higher vulnerability to neuropsychiatric disorders (Moon et al., 2018). Preclinical findings showed that selective deletion of CACNA1 in serotonergic neurons decreased active-coping and increased passive-coping behavior in the FST, a behavioral effect that was abrogated by treatment with a 5-HT1A receptor antagonist. Also, CACNA1C knockout increased the activation of serotonergic neurons in the caudal DRN (Ehlinger and Commons, 2019).

**Figure 4:**
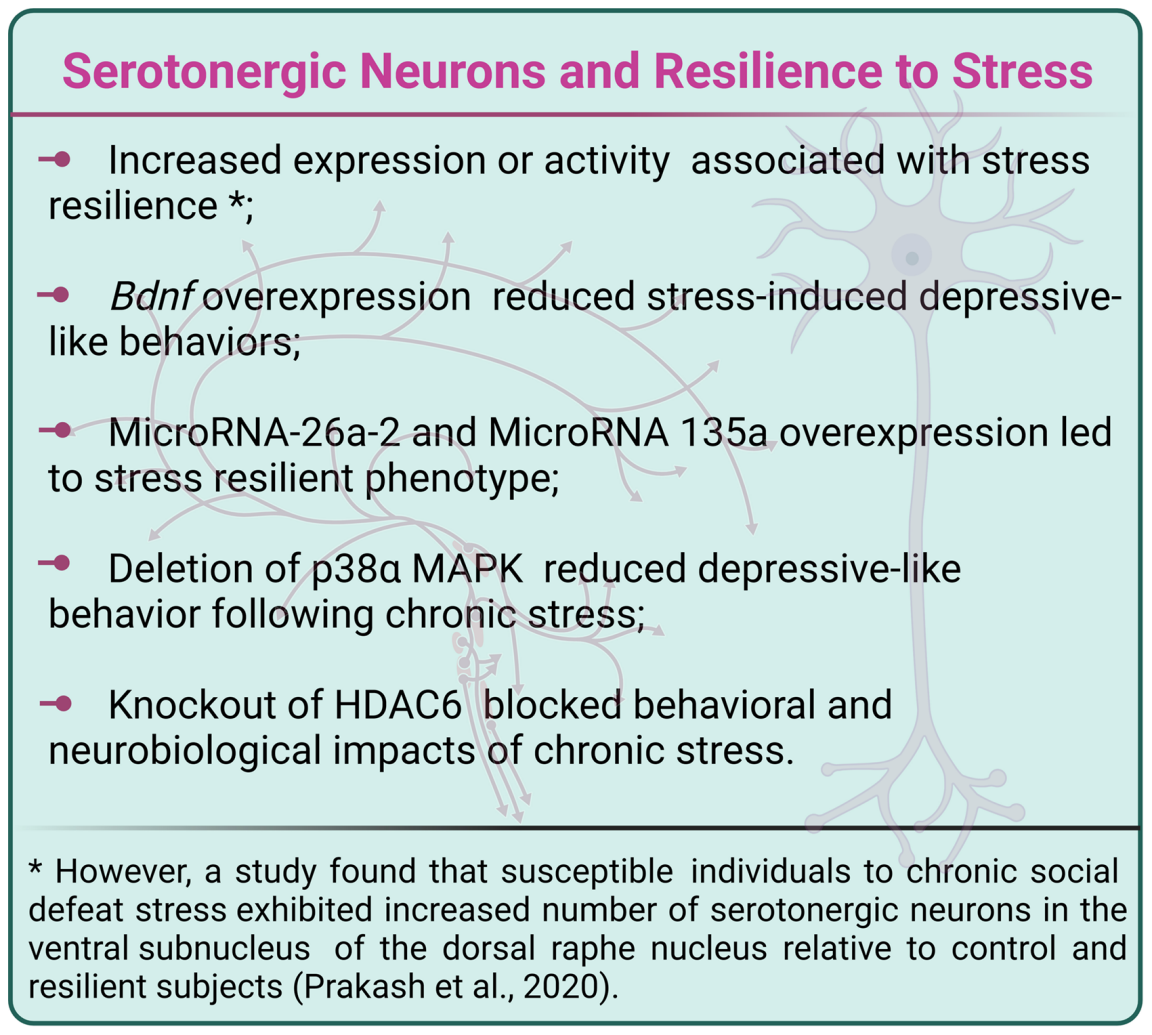
Manipulation of *Bdnf*, microRNAs 26a-2 and 135a, p38α MAPK and HDAC6 within serotonergic neurons induced a stress-resilient phenotype. Created with BioRender.com.

Finally, specific mitogen-activated protein kinases (MAPKs) and enzymes that regulate epigenetic processes seem to be part of the molecular machinery that regulates serotonergic neuron activity toward stressful events, leading to responses related to stress resilience or susceptibility. The p38 MAPKs represent one of the four subgroups of MAPKs which pathway is activated by several extracellular stimuli, including mitogens, irradiation, heat shock, and proinflammatory cytokines (Obata et al., 2000). Ultimately, p38 pathway triggering has been associated with inflammation, cell cycle, development, and apoptosis processes, among others (Zarubin and Han, 2005). Mice presenting deletion of the p38α MAPK in serotonergic neurons showed increased social investigation and reduced immobility in the FST following SDS, in comparison to controls. Also, p38α MAPK deletion in serotonergic neurons prevented the reinstatement of cocaine-seeking behavior induced by stress (Bruchas et al., 2011). In addition, the epigenetic enzymes histone deacetylases (HDACs) remove acetyl groups from histones, which promote chromatin condensation and consequent inhibition of gene expression. Espallergues et al. (2012) demonstrated that HDAC6 is involved in neuroplasticity mediated by glucocorticoid receptors in serotonergic neurons. Interestingly, selective knockout of HDAC6 in serotonergic neurons inhibited social avoidance, hypoexcitability, and hypertrophy of serotonergic neurons following SDS (Espallergues et al., 2012; Figure 4).

### 3.5. Involvement of other interneuron subtypes

In addition to the previously discussed important involvement of PV interneurons, other interneuron subpopulations also seem to be implicated in resilience and vulnerability processes. Here, we very briefly exemplify some of the other interneuron subtypes involved in those distinct stress-related responses to help the reader on interpreting results involving PV interneurons manipulation, and, for a complete review, we refer the reader to the works by McKlveen et al. (2019) and Albrecht et al. (2021).

For example, chemogenetic inhibition of cholinergic interneurons in the NAc produced a susceptible phenotype in mice subjected to SSDS, characterized by reduced social interaction, preference for palatable sucrose solution, and increased immobility time in the tail suspension and FST. Accordingly, repeated activation of accumbal cholinergic interneurons reversed social interaction deficits in mice susceptible to CSDS (Cheng et al., 2019).

Regarding the somatostatin-expressing subpopulation, susceptible subjects to chronic stress exhibited a reduced number of somatostatin interneurons in the primary motor cortex (Serradas et al., 2022) and DG, CA1, and CA2-3 areas of the ventral HIP (Czéh et al., 2015) relative to resilient animals. Disinhibition of somatostatin-expressing GABAergic interneurons produced by selective deletion of γ2 GABAA receptors promoted a resilient phenotype to CUMS, in male but not female mice, in the novelty-suppressed feeding test, and prevented stress-induced loss of mPFC somatostatin neurons in both sexes (Jefferson et al., 2020). In addition, the ablation of mGlu5 receptors from somatostatin interneurons in the mPFC prevented the impacts of exposure to acute restraint stress on specific cognitive behaviors (Joffe et al., 2022).

Czéh et al. (2015) observed a decrease in the number of calretinin-positive interneurons in the CA1 area of the dorsal HIP and of NPY interneurons in CA1 and CA2-3 regions of ventral HIP, in stress susceptible relative to resilient and control rats (Czéh et al., 2015). Another study also found augmented activation of NPY interneurons in the dorsal DG in rats classified as resilient to a protocol of juvenile stress combined with trauma exposure in adult life (Regev-Tsur et al., 2020). Accordingly, the knockdown of NPY in the dorsal DG reduced the prevalence of resilient subjects to this posttraumatic stress disorder model (Regev-Tsur et al., 2020).

Although cholecystokinin-positive (CCK+) interneurons seem to be affected by stress exposure and implicated in stress-related outcomes, available data is still inconclusive due to mixed results. Susceptible rats to a model of posttraumatic stress disorder presented an increase in the activation of CCK+ interneurons in the ventral DG and basolateral amygdala, relative to control and resilient animals (Regev-Tsur et al., 2020). The CMS exposure induced a decrease in CCK+ interneurons in the PL and IL mPFC of both resilient and susceptible rats (Czéh et al., 2015), while early-life stress exposure decreased the number of CCK+ neurons in the medial orbitofrontal cortex (Poleksic et al., 2021). On the other hand, there is a study showing that the administration of the CCKB receptor antagonist in the mPFC induces a resilient phenotype to CSDS (Vialou et al., 2014), while another showed an increase in the number of CCK+ interneurons in the ventral orbitofrontal cortex of resilient rats to CMS (Varga et al., 2017).

## 4. Concluding remarks

In this review, we approached how innovative scientific techniques have helped researchers to unravel the role of non-neuronal and neuronal cell subpopulations of different brain areas in stress-induced outcomes, resilience, and vulnerability. Also, works have shed light on how diverse molecular mechanisms, involving specific receptors, neurotrophic factors, epigenetic enzymes and miRNAs within these brain cell subtypes are crucial for modulating their activity and associate with the expression of a stress-susceptible or resilient phenotype, advancing even more our understanding of this complex and multifactorial process. Beyond that, it is of absolute importance that further studies explore how stress differently affects the communication of these different brain cell subpopulations within neural circuits in susceptible versus resilient individuals, leading to diverse physiological and behavioral responses. Also, it would be extremely valuable to delve into sexes differences and the mechanism of resilience and susceptibility in females, once most studies failed to further investigate this particularity.

## Author contributions

CF, MP, and GM-S performed the literature research and wrote the manuscript. All authors contributed to the manuscript revision and approved the submitted version.

## Funding

This work was supported by grants 2020/15216-2 and 2021/13291-0 from São Paulo Research Foundation (FAPESP) to GM-S, 2019/24073-3 and 2022/10168-5 from São Paulo Research Foundation (FAPESP) to CF, and 2020/08363-9 from São Paulo Research Foundation (FAPESP) to MP.

## Conflict of interest

The authors declare that the research was conducted in the absence of any commercial or financial relationships that could be construed as a potential conflict of interest.

## Publisher’s note

All claims expressed in this article are solely those of the authors and do not necessarily represent those of their affiliated organizations, or those of the publisher, the editors and the reviewers. Any product that may be evaluated in this article, or claim that may be made by its manufacturer, is not guaranteed or endorsed by the publisher.
